# Cross‐education attenuates muscle weakness and facilitates strength recovery after orthopedic immobilization in females: A pilot study

**DOI:** 10.14814/phy2.70329

**Published:** 2025-04-26

**Authors:** Joshua C. Carr, Caleb C. Voskuil, Justin W. Andrushko, Rob J. MacLennan, Jason M. DeFreitas, Matt S. Stock, Jonathan P. Farthing

**Affiliations:** ^1^ Department of Kinesiology Kansas State University Manhattan Kansas USA; ^2^ Department of Kinesiology Texas Christian University Fort Worth Texas USA; ^3^ Department of Medical Education Texas Christian University School of Medicine Fort Worth Texas USA; ^4^ Department of Exercise Science Lakeland University Plymouth Wisconsin USA; ^5^ Department of Sport, Exercise and Rehabilitation, Faculty of Health and Life Sciences Northumbria University Newcastle upon Tyne, Tyne and Wear UK; ^6^ Department of Neurology University of Florida Gainesville Florida USA; ^7^ Malcom Randall Department of Veterans Affairs Medical Center Brain Rehabilitation Research Center Gainesville Florida USA; ^8^ Department of Exercise Science Syracuse University Syracuse New York USA; ^9^ University of Central Florida, School of Kinesiology and Rehabilitation Sciences Orlando Florida USA; ^10^ University of Saskatchewan, College of Kinesiology Saskatoon Saskatchewan Canada

**Keywords:** early medical intervention, physical therapy, resistance training, sports medicine

## Abstract

This pilot study consists of a two‐phase intervention to examine the effectiveness of unilateral resistance training to mitigate the negative consequences of immobilization and expedite the restoration of muscle strength and size following a period of retraining. Ten females were randomized to a unilateral training (TRAIN, *n* = 6) or control (CON, *n* = 4) group. During Phase 1, all participants wore an arm sling for a total of 4 weeks on their non‐dominant arm. This phase required the TRAIN group to perform unilateral resistance training with the non‐immobilized arm while the CON group did not. Phase 2 commenced thereafter and consisted of 4 weeks of bilateral resistance training for both groups. Outcome measures of neuromuscular function and muscle size were assessed at baseline and after each phase, with neuromuscular function quantified by maximal dynamic and isometric strength alongside electromyographic responses and muscle size measured using ultrasonography and regional lean mass via DEXA. Unilateral training of the non‐immobilized arm during Phase 1 attenuated dynamic (*p* < 0.05; *g* > 1.2), but not isometric (*p* > 0.40; *g* < 0.095), strength loss following immobilization and showed large effects for improving the recovery of strength after retraining. Similarly, the imaging data show the relative changes in muscle size and regional lean mass of the non‐dominant arm favor TRAIN. Although the small sample prevents definitive conclusions, our study suggests resistance training of the non‐immobilized arm attenuates muscle weakness and atrophy for the contralateral, immobilized arm during immobilization and facilitates their recovery following retraining.

## 1 INTRODUCTION

Orthopedic immobilization is a necessary part of the rehabilitation process following common sports‐related injuries and surgical interventions. These injuries typically affect one side of the body and often result in temporary muscle disuse due to the indicated immobilization. During this time, however, detrimental changes occur along the motor pathway that precipitate muscle weakness, skeletal muscle atrophy, and functional impairments in descending neural drive before the prescribed length of immobilization is complete (Campbell et al., 2019). Emerging evidence shows that resistance training of the opposite, unaffected limb while undergoing immobilization attenuates the undesirable changes in neuromuscular function (Andrushko et al., 2018). This cross‐limb phenomenon derives from the cross‐education effect, where resistance training of one limb confers neural adaptations that serve to improve strength for the contralateral, untrained limb (Farthing & Zehr, 2014; Manca et al., 2021). Given the prevalence of orthopedic injuries that lead to immobilization and disuse, accessible countermeasures such as unilateral training offer a means by which individuals can maintain and even improve muscle fitness during an otherwise profound period of inactivity.

The preservation of motor function for the immobilized limb following cross‐education is likely mediated through redundant and diffusive cross‐limb motor networks within the descending pathways of the central nervous system (Glover & Baker, 2020; Manca et al., 2018). Despite the theoretical framework proposed by the restoring symmetry hypothesis (Farthing & Zehr, 2014) and recent calls for the prescription of cross‐education within standard care treatments for orthopedic injuries (Collins et al., 2017; Voskuil, Andrushko, et al., 2023), the use of cross‐education as a complementary approach to facilitate recovery for the affected (immobilized) limb has largely been ignored. At present, seven studies (Andrushko et al., 2017; Chen et al., 2023; Farthing et al., 2009; Farthing et al., 2011; Magnus et al., 2010; Pearce et al., 2013; Valdes et al., 2021) have examined the influence of cross‐education on the immediate recovery outcomes for the opposite immobilized limb in healthy participants. These experiments largely support the restoring symmetry hypothesis by observing an attenuation of muscle weakness and atrophy versus immobilization‐only control groups immediately following immobilization (Andrushko et al., 2017; Chen et al., 2023; Farthing et al., 2009; Farthing et al., 2011; Magnus et al., 2010; Pearce et al., 2013; Valdes et al., 2021). What has yet to be addressed, however, is the timeline of the immobilized limb's response to retraining. This is a critical piece of evidence needed to bolster the restoring symmetry hypothesis and further the clinical applications of cross‐education. It stands to reason that the evidence showing that cross‐education ameliorates muscle weakness due to immobilization should then enhance the return of muscle strength and limb symmetry, yet this is currently unknown. Resolving this issue in non‐clinically indicated paradigms of muscle unloading is an important step to bridge the recent debate over the utility of cross‐education in post‐surgical rehabilitation (Andrushko et al., 2023; Kotsifaki et al., 2023).

The paradigms employed to study the preservation effects of cross‐education have been performed mostly with arm slinging for a period of 21–28 days (Chen et al., 2023; Farthing et al., 2009; Pearce et al., 2013; Valdes et al., 2021). During this time, maximal strength losses between ~10% and 20% have been reported, with losses of muscle thickness ranging from ~2% to 6% (Andrushko et al., 2018). Immobilization precipitates the greatest rate of maladaptation on neuromuscular function during the early phases of muscle unloading (Campbell et al., 2019). Being so, the rationale for administering cross‐education once immobilization commences is clear based on recent evidence showing the attenuation of neuromuscular dysfunction once immobilization is complete (Andrushko et al., 2018). However, the critical issues concerning the detraining–retraining timeline remain unresolved. This pilot study addresses this gap by evaluating the efficacy of cross‐education to support strength recovery during retraining, providing preliminary proof‐of‐concept data to support future larger‐scale trials. Assessments were conducted at baseline, after 4 weeks of immobilization, and after 4 weeks of retraining. Neuromuscular function was measured via maximal dynamic strength (biceps curl and shoulder press 1RM) and maximal voluntary isometric contraction (MVC) of the elbow flexors, alongside electromyographic (EMG) responses of the biceps brachii. Muscle size and quality were measured using ultrasonography‐derived muscle cross‐sectional area (mCSA) and echo intensity (cEI), with regional lean mass assessed via Dual‐Energy X‐ray Absorptiometry (DXA). We hypothesized that cross‐education would reduce immobilization‐induced neuromuscular deficits and facilitate recovery during retraining.

## 2 METHODS

### 2.1 Experimental design

**FIGURE 1:**
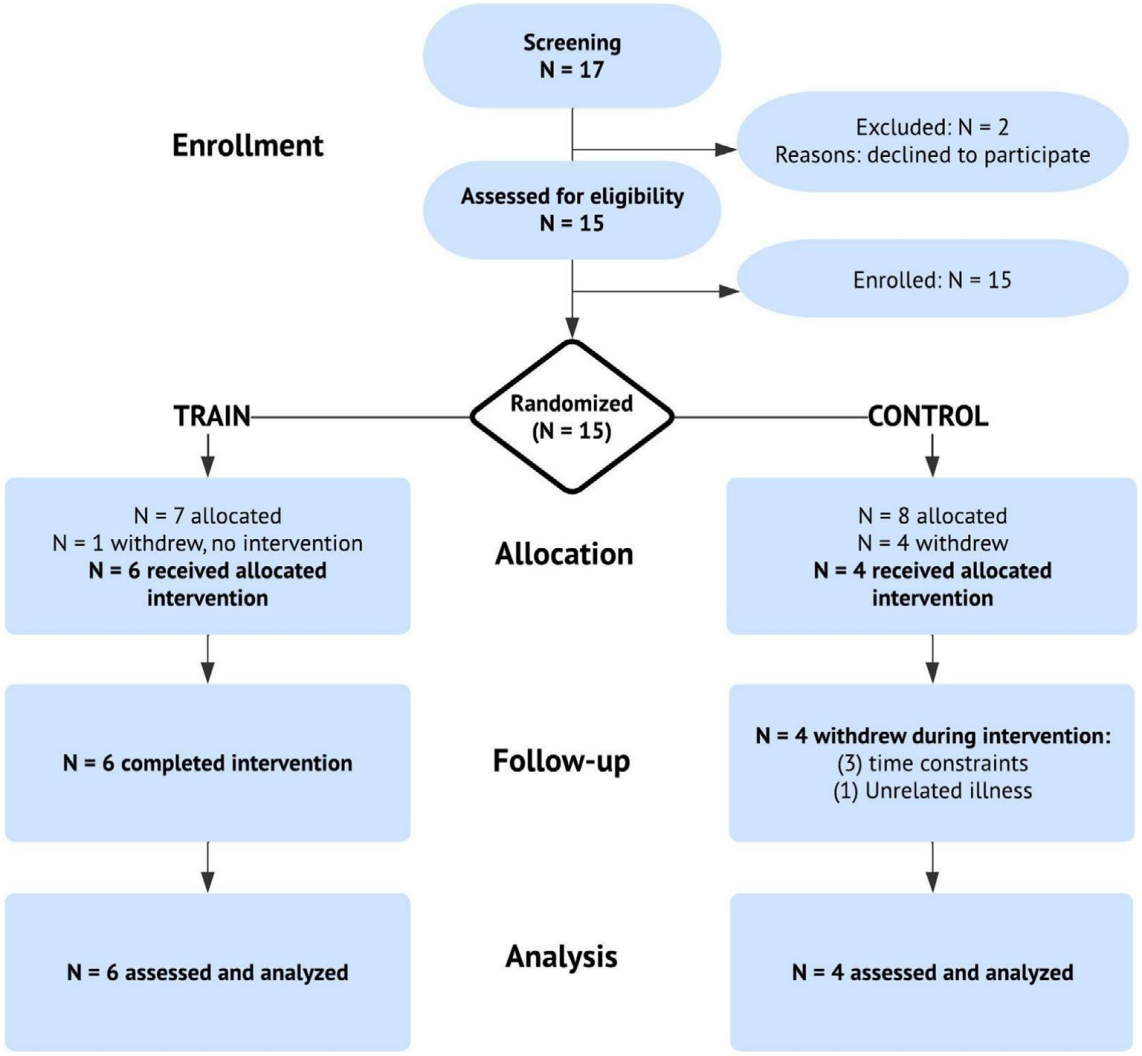
The CONSORT flow diagram for the complete trial is shown.

A randomized controlled study design examined the effects of arm slinging with and without concomitant cross‐education training. Participants were randomly assigned to the cross‐education intervention group (TRAIN) or the immobilization‐only control group (CON). The 10‐week study consisted of two phases. In Phase 1, both TRAIN and CON underwent 4 weeks of non‐dominant arm immobilization. During this period, TRAIN performed progressive resistance training of the contralateral, dominant arm, while CON did not. Phase 2 comprised 4 weeks of bilateral strength retraining for all participants. The outcome measures were captured at baseline and after each phase. This study was approved by the Institutional Review Board for Human Subjects Research at Texas Christian University (#2021–101) and adhered to the principles of the Declaration of Helsinki (ClinicalTrials.gov identifier: NCT05097092). Participants provided written Informed Consent before enrollment. The CONSORT (Eldridge et al., 2016) flow diagram is shown in Figure 1.

### 2.2 Study participants

The baseline demographics for the participants are shown in Table 1. A total of 10 participants (*n* = 10 females) completed the study in its entirety, with four in the control group and six in the experimental group. We performed an a priori power analysis with G*Power software using an effect size (*f* = 0.40) for maximal strength changes based on similar within‐between mixed designs (Magnus et al., 2010; Valdes et al., 2021). The power analysis computed that a total of 16 participants would be required to be adequately powered. The inclusion criteria were a body mass index between 18 and 35 kg/m^2^, 18 and 35 years of age, and right‐hand dominance. The exclusion criteria were a personal or familial history of blood clots, orthopedic surgeries, or injuries to the upper limbs within the last 6 months, pregnancy and/or nursing, and resistance training within the last 3 months.

**TABLE 1:** Participant demographics and baseline descriptive statistics showing means ± standard deviations.

Demographic	TRAIN	CON
*N*	6F	4F
Age (yr)	19 ± 0.5	19 ± 0.5
Height (cm	165.9 ± 10.1	159.4 ± 5.6
Mass (Kg)	58.7 ± 9.1	60.4 ± 13.9
Hormonal contraceptive use	5/6	3/4
Body fat (%)	36.0 ± 5.7	28.6 ± 4.6
Handedness	Strong right 6/6	Strong right 4/4
Shoulder press 1RM (Kg)	11.7 ± 1.9	9.8 ± 1.6
Bicep curl 1RM (Kg)	9.1 ± 2.0	8.1 ± 1.5
Elbow flexor MVC (N)	194.6 ± 27.2	174.1 ± 27.8
Handgrip strength (Kg)	29.7 ± 4.4	25.5 ± 3.1

*Note*: Participant characteristics by group are listed. Hormonal contraceptive use, assessed via menstrual history forms, is reported as number of users per group. Body fat percentage was measured using dual‐energy‐X‐ray absorptiometry at baseline. Non‐dominant hand grip strength at baseline and handedness (assessed with the Waterloo handedness questionnaire) are also reported. No significant differences (*p* > 0 0.05) were observed between groups for any measure.

### 2.3 Orthopedic immobilization

Immobilization was implemented using a sling and swathe on the non‐dominant arm, similar to previous orthopedic disuse models (Magnus et al., 2010; Pearce et al., 2013; Valdes et al., 2021). Participants wore the sling for 28 days during waking hours, aiming for 10 h per day, with removal allowed for bathing, sleeping, and driving. The sling kept the arm at 90° flexion, while the swathe secured it to the body. Participants received training on performing daily tasks with the sling and reported their wearing hours via an online survey. To prevent adverse effects, they were instructed to perform ~3 min of shoulder mobility exercises daily and were assigned a safety officer for daily check‐ins and reminders. In total, the self‐reported time spent wearing the sling and swathe was 10.4 ± 1.6 h per day with no significant differences between groups.

### 2.4 Training intervention

This study included two resistance training periods: Phase 1, exclusive to the TRAIN group, and Phase 2, involving both TRAIN and CON groups. The TRAIN group completed eight unilateral resistance training sessions in Phase 1, while both groups completed eight resistance training sessions in Phase 2 involving both arms. All training was supervised by a Certified Strength and Conditioning Specialist, with the same researcher guiding participants throughout the study. The interventions included dumbbell biceps curls and shoulder presses, starting at 75% of each participant's 1RM, with three sets of five repetitions. Progressive overload was implemented by increasing sets to five across the first three sessions of each phase, with intensity increasing as tolerated. To reduce muscle soreness and edema, volume was reduced to three sets for the final two sessions of each phase (Magnus et al., 2010). In Phase 2, participants consumed lactose‐free cow's milk (~0.2 g/lb. protein) after each session to support muscle recovery, similar to others (Pagan et al., 2024; Stock et al., 2017). Expanded details regarding the immobilization and training interventions are provided in Appendix S1.

### 2.5 Instrumentation and testing procedures

A standardized familiarization protocol for strength testing was conducted during the first lab visit, following screening, consent, and imaging. Strength testing followed an isometric‐then‐dynamic order, starting with biceps curls followed by shoulder press, with 90‐s rest intervals between attempts. Arm testing order was randomized, except post‐immobilization, when the non‐immobilized limb was tested first. Maximal isometric and dynamic strength assessments occurred approximately 3 days after familiarization.

### 2.6 Maximal strength and EMG assessment

Handgrip strength was obtained with a Jamar handgrip dynamometer (Paterson Medical, Green Bay, WI, USA) and grip span was noted and maintained for each participant. Then, isometric elbow flexion strength was measured while participants were seated with elbows at 90° via a restrained tension‐compression load cell (Model SSM‐AJ‐500, Interface, Inc., Scottsdale, AZ). Participants performed three unilateral MVCs within a 5% range, with verbal and visual cues. Dynamic strength was assessed via unilateral bicep curls and shoulder presses, determining 1RM using adjustable dumbbells. 1RM testing was completed within five attempts, using 0.25lb microplates when necessary for precise evaluation. Researchers recorded each attempt, and participants rated their exertion using the OMNI scale to guide load adjustments. Briefly, bipolar surface EMG activity was collected from the biceps brachii during strength testing (Delsys Inc., Natick, MA, USA.) according to established standards. The maximal isometric force and EMG amplitude (EMG_AMP_) were defined as the peak value of the filtered signals within 500 ms and 100 ms windows, respectively, with the signal processing procedures as described elsewhere (Augsburger et al., 2022). The maximal values from the three attempts during the respective testing visit and task were then averaged and used for statistical analysis and also normalized against the respective baseline values. Isometric force and EMG signal analyses were performed by a researcher blind to group allocation.

### 2.7 B‐mode ultrasonography & DXA imaging

B‐mode ultrasonography (GE LOGIQ E10; GE Healthcare, Milwaukee, WI, USA) was used to measure mCSA and subcutaneous‐corrected echo intensity (cEI) of the biceps brachii. The ultrasonography images were collected with a wideband linear array probe (GE L8‐18i‐RS, 4.5–18 MHz, 25mm field of view; GE Healthcare, Milwaukee, WI, USA). The settings were held consistent (Frequency: 12 Hz, Gain: 55 dB, Dynamic range: 75) between each participant and timepoint (Carr et al., 2021; Jenkins et al., 2015) with the same acquisition and analysis procedures as described previously from our group (Carr et al., 2021; Voskuil, Dudar, et al., 2023) and elsewhere (Jenkins et al., 2015; Young et al., 2015) to capture mCSA at 50% of the distance from the acromion process to the antecubital space. Our laboratory has demonstrated strong test–retest reliability for mCSA measurements of the biceps brachii (ICC >0.90) (Carr et al., 2021). A full body DXA scan (GE Lunar iDXA) was performed on each participant at the specified timepoints. We used DXA to obtain regional lean body mass (RegLBM; lb) for the left side of the upper body. The regional body composition results were obtained by region of interest (ROI) analyses based on the manufacturer's settings that encompassed the tissue from the lateral fifth cervical and fifth lumbar vertebrae. We also obtained total body fat percentage (BF%). All images were coded and analyzed by a member of the research team who was blinded to the participant, group allocation, and time point of the respective ultrasound and DXA imaging.

### 2.8 Statistical analysis

The primary outcome variables are the maximal strength values of the immobilized arm assessed via biceps curl and shoulder press 1RM, as well as maximal isometric strength of the elbow flexors and handgrip. Additional outcome measures include EMG amplitude of the biceps brachii during 1RM and MVC, mCSA and cEI of the biceps brachii, as well as RegLBM and total BF%. One‐tailed independent samples *t*‐tests with Hedge's *g* effect size statistics were used to examine the relative changes at each phase to identify mean differences between groups related to our hypotheses. This approach was selected over omnibus tests due to the small sample size and due to its focus on the specific changes between groups at each phase relative to baseline, allowing for a more straightforward interpretation of the differences without the assumptions and complexity involved in modeling interaction effects across all time points. By examining the change scores directly, the analysis provides a clearer view of the effect sizes and practical significance of changes within each phase, aligning more closely with our aims to pinpoint the precise points of divergence between groups. Exploratory analyses of strength gains and morphological changes in the immobilized arm are shown in Appendix S1. All data were analyzed with JASP Software (JASP Team, 2024) and reported as means ± SD. *α* was set at 0.05.

## 3 RESULTS

### 3.1 Maximal strength & EMG_AMP_ activity

In the biceps curl, TRAIN lost significantly less strength than CON following immobilization (+5.81 ± 14.6% vs. −12.6 ± 9.3%, *p* = 0.029, *g* = 1.29), though this difference was not significant after retraining (+24.1 ± 13.3% vs. +12.3 ± 11.5%, *p* = 0.094, *g* = 0.839). Similar trends were observed in the shoulder press: TRAIN lost less strength than CON post‐immobilization (+13.9 ± 24.1% vs. −13.1 ± 9.14%, Mann–Whitney *p* < 0.01, *g* = 1.00), but there was no significant difference after retraining (+34.9 ± 25.0% vs. +21.7 ± 13.3%, *p* = 0.184, *g* = 0.556). Elbow flexor MVC and handgrip strength showed no significant differences between groups in both phases (*p* > 0.4).

**FIGURE 2:**
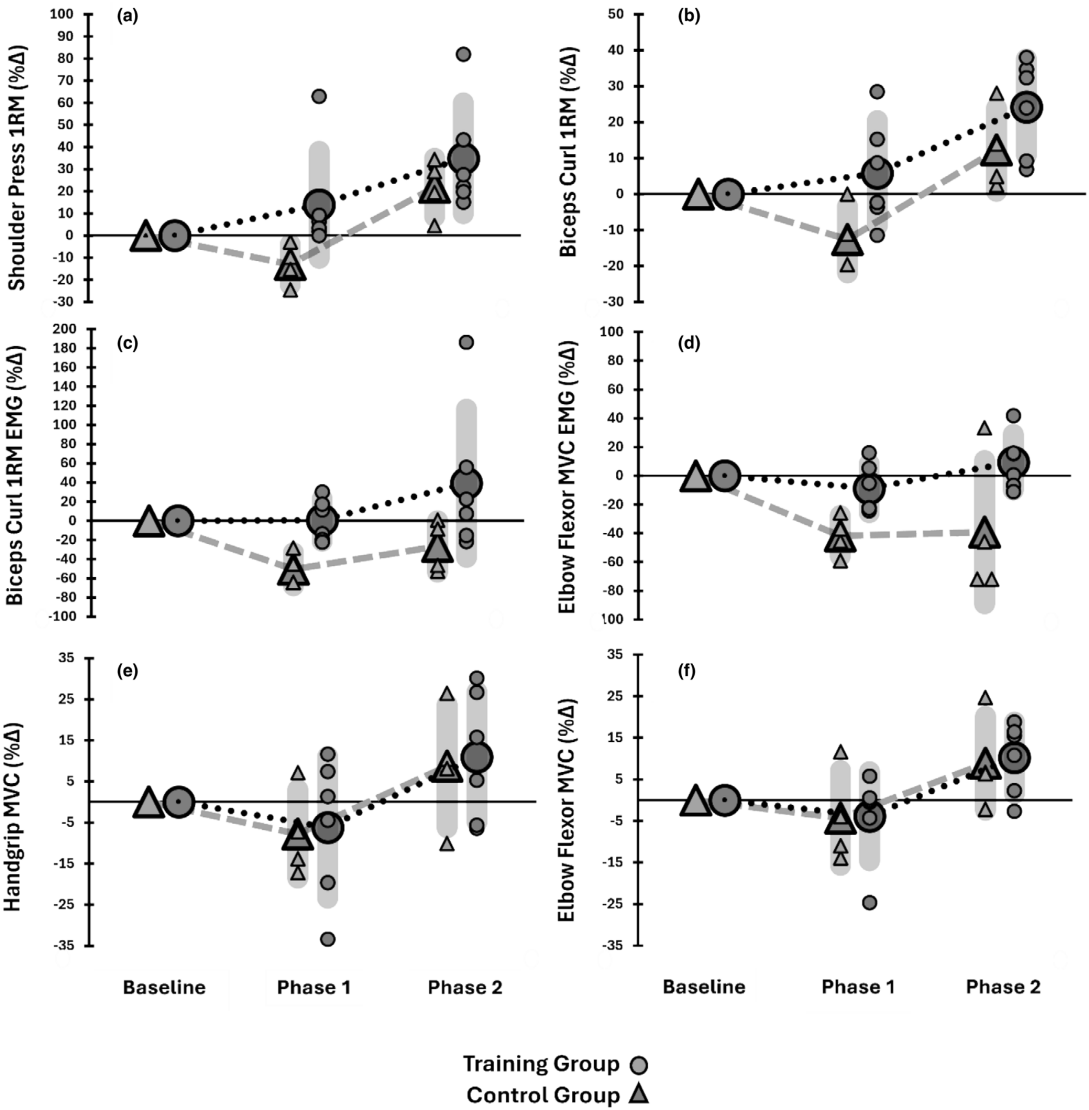
The relative changes in the immobilized arm for shoulder press 1RM (a), biceps curl 1RM (b), biceps curl 1RM EMG_AMP_ (c), elbow flexor MVC EMG_AMP_ (d), handgrip MVC strength (e), and elbow flexor MVC strength (f) from baseline for TRAIN (circles) and CON groups (triangles) are shown. The large symbols represent the group mean, with the standard deviation shown in the gray shading, along with the individual responses after Phase 1 (immobilization) and Phase 2 (retraining).

For the EMG_AMP_ response, TRAIN retained significantly more 1RM EMG_AMP_ than CON post‐immobilization (+0.59 ± 21.9% vs. −50.5 ± 17.2%, *p* = 0.002, *g* = 2.27), but this was not significant after retraining (+39.1 ± 77.2% vs. −26.8 ± 26.8%, *p* = 0.072, *g* = 0.942). TRAIN also lost less MVC EMG_AMP_ post‐immobilization (−8.8 ± 16.9% vs. −41.8 ± 14.0%, *p* < 0.01, *g* = 1.87) and had greater MVC EMG_AMP_ after retraining (+9.3 ± 19.3% vs. −39.2 ± 49.8%, *p* = 0.029, *g* = 1.28). The strength and EMG_AMP_ data for the immobilized arm are shown in Figure 2.

### 3.2 Ultrasonography & DXA imaging

For biceps brachii mCSA, TRAIN lost significantly less mCSA than CON post‐immobilization (+1.10 ± 5.23% vs. −5.32 ± 4.8%, *p* = 0.043, *g* = 1.13), though this difference was not significant after retraining (+8.1 ± 2.7% vs. +4.5 ± 4.7%, *p* = 0.082, *g* = 0.894). CON had significantly greater increases in mCSA cEI than TRAIN post‐immobilization (+11.7 ± 15.50% vs. −8.0 ± 10.8%, *p* = 0.039, *g* = 1.33), but this was not significant after retraining (+4.0 ± 17.4% vs. −5.8 ± 7.2%, *p* = 0.123, *g* = 0.731).

**FIGURE 3:**
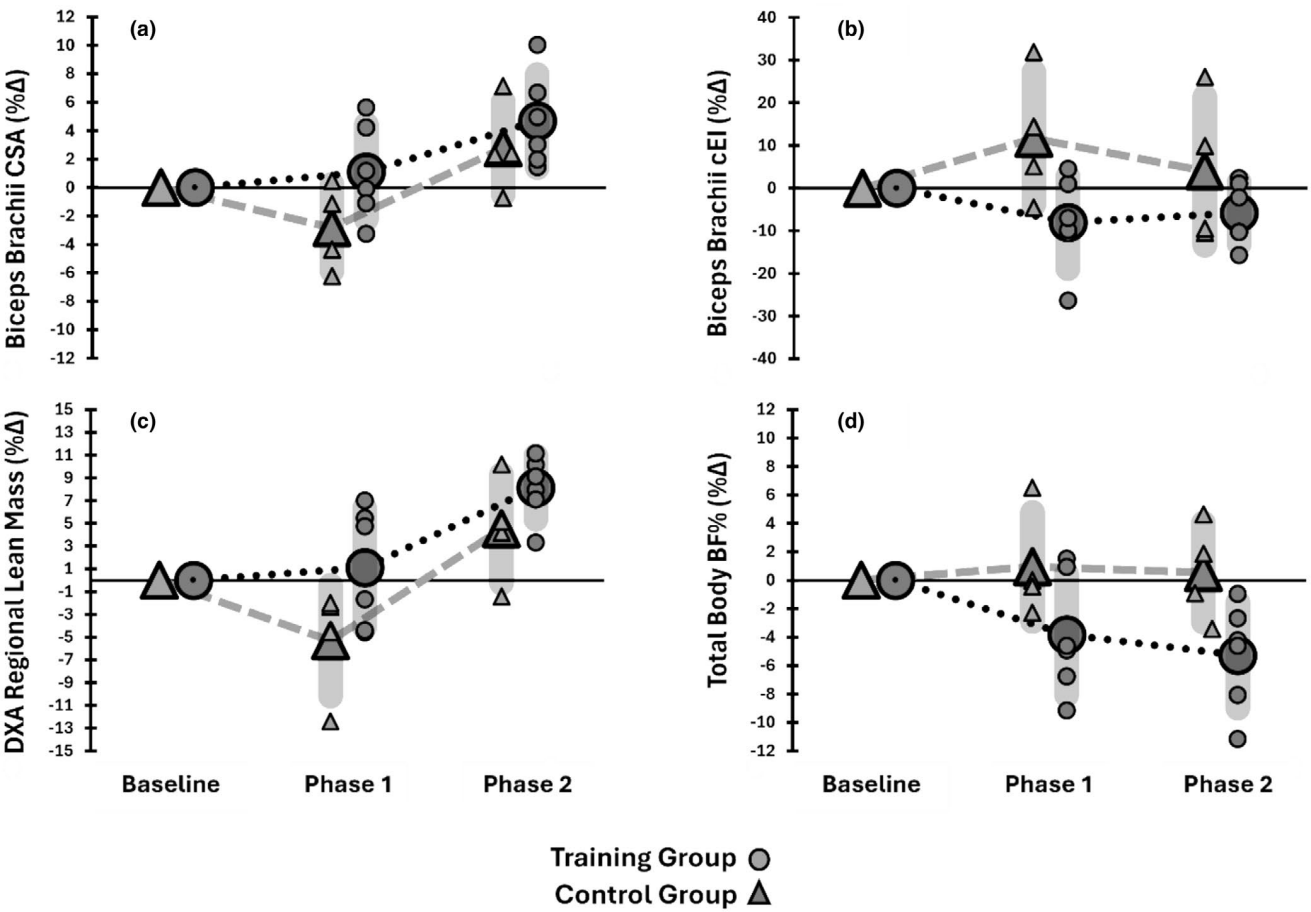
The relative changes in biceps brachii CSA (a), biceps brachii cEI (b), regional lean mass (c), and total body BF% (d) from baseline for TRAIN (circles) and CON groups (triangles) are shown. The large symbols represent the group mean, with the standard deviation shown in the gray shading, along with the individual responses after Phase 1 (immobilization) and Phase 2 (retraining).

For RegLBM, CON had greater decreases than TRAIN post‐immobilization (−2.8 ± 3.0% vs. +1.09 ± 3.3%, *p* = 0.048, *g* = 1.09), but differences were not significant after retraining (+2.7 ± 3.2% vs. +4.6 ± 3.2%, *p* = 0.195, *g* = 0.530). Body fat% was not significantly different post‐immobilization (+0.94 ± 3.8% vs. −3.8 ± 4.2%, *p* = 0.055, *g* = 1.05), but TRAIN had significantly greater reductions than CON post‐retraining (−5.2 ± 3.7% vs. +0.54 ± 3.4%, *p* = 0.019, *g* = 1.44). The imaging data are shown in Figure 3.

## 4 DISCUSSION

The purpose of this study was to examine whether cross‐education attenuates losses in muscle function during orthopedic immobilization and subsequently facilitates the recovery of strength following a period of strength retraining. The novel findings of this small preliminary study demonstrate, for the first time, that cross‐education likely facilitates the recovery of muscle strength in a task‐specific manner after the previously immobilized limb undergoes a period of strength retraining. Although our study is too small to offer definitive conclusions, our observations of strength and size preservation following immobilization largely support the restoring symmetry hypothesis and the previous data on this topic (Magnus et al., 2010; Pearce et al., 2013; Valdes et al., 2021; Farthing et al., 2009; Andrushko et al., 2017; Chen et al., 2023). Importantly, our findings offer further support for the preservation of skeletal muscle size and lean mass during disuse with unilateral resistance training (Andrushko et al., 2017; Chen et al., 2023; Magnus et al., 2010; Valdes et al., 2021) and extend the implications of the cross‐education paradigm in restoring muscle size once the immobilized limb is able to undergo resistance training.

The experiments that have used cross‐education to attenuate immobilization‐induced deficits have primarily used the upper limb with an unloading period of three (Farthing et al., 2009; Pearce et al., 2013; Chen et al., 2023; Farthing et al., 2011) or 4 weeks (Andrushko et al., 2017; Magnus et al., 2010; Valdes et al., 2021). While Magnus et al. (2010) trained maximal isometric elbow flexion and extension during immobilization, a unique aspect of the present study as it relates to cross‐education and detraining paradigms is the use of two multi‐joint, compound tasks. The relevance of this lies in the cumulative loading imposed on the central nervous system and the pragmatism that cross‐education paradigms offer during a period of muscle unloading. Most studies (Farthing et al., 2009; Pearce et al., 2013; Valdes et al., 2021; Andrushko et al., 2017; Farthing et al., 2011) involve groups of males and females, while only one study used all male participants (Chen et al., 2023). Notably, all participants in the present study were female. This is particularly relevant because females are more susceptible to disuse‐induced loss of muscle size and strength (Girts et al., 2024; Michel et al., 2024; Trappe et al., 2023). Therefore, our findings provide novel evidence that cross‐education can serve as an effective mitigation strategy during disuse in females, a group that is both understudied and more vulnerable to disuse‐induced impairments (Girts et al., 2024; Michel et al., 2024; Trappe et al., 2023). We suggest these data are important in the literature as they can inform future meta‐analyses examining sex differences in response to cross‐education and disuse paradigms.

In the context of a training intervention, it is important to consider the relevance of assessing strength outcomes that were or were not specific to the training intervention itself (Buckner et al., 2017; Pagan et al., 2024). Our outcome measurements of strength were assessed with 1RM of the unilateral shoulder press and biceps curl along with maximal isometric strength of the elbow flexors and handgrip to provide a robust view of the training‐specific and non‐specific impairments and improvements of maximal strength with our detraining‐retraining paradigm. Most of the previous studies have tested the strength of the trained task (Farthing et al., 2009; Magnus et al., 2010; Andrushko et al., 2017; Farthing et al., 2011), while some have tested additional strength outcomes like maximal isometric strength of the elbow flexors following training with dynamic biceps curl (Chen et al., 2023; Pearce et al., 2013), or included specific and non‐specific contraction types (Andrushko et al., 2017). Pearce et al. (2013) show that after their immobilization intervention, the immobilization‐only control group experienced a −19.9% loss of 1RM strength and a −5.7% loss of MVC strength, whereas the cross‐education group showed little evidence of strength loss during 1RM and slight improvement in MVC force of +2.7%. Similarly, Magnus et al. (2010) show the cross‐education group improved strength of the immobilized limb by +7.7% and +32.2% during elbow flexion and extension, respectively, while the control group observed a change of +4% and −6.1% of those outcomes following the intervention. More recently, the interventions (Chen et al., 2023; Valdes et al., 2021) testing the influence of cross‐education training with eccentric emphasis show *improvements* in average strength changes following immobilization. Following eccentric training, Chen et al. (2023) report a +3.3% increase in MVC force and Valdes et al. (2021) show improvements of +2.5% and +11.7% for 1RM and MVC strength of the immobilized arm, whereas the control groups experienced declines in maximal strength of their immobilized arm in the magnitudes of −16.7% and −12.3% (Chen et al., 2023) and −21.5% (Valdes et al., 2021), respectively. We show that immediately following training, the immobilized arm of TRAIN increased by ~13% for shoulder press and ~4% for biceps curl 1RM, whereas CON lost an average of −13% in their maximal dynamic strength. However, both groups appear to show similar patterns of strength loss and gain for isometric measurements of strength. The similar patterns of strength change for elbow flexion MVC are interesting when compared to Pearce et al. (2013) who show isometric strength preservation following dynamic training and also within the context of the present EMG data. Here, we show the normalized EMG of the biceps brachii was diminished by ~40% for CON following immobilization, whereas TRAIN shows ~8% loss, with similar patterns following retraining.

Following a period of immobilization with an arm sling, the relative change in muscle thickness of the biceps brachii decreases by ~3%–5% (Magnus et al., 2010; Pearce et al., 2013), and recent data (Chen et al., 2023) show a loss of biceps brachii mCSA by ~12%. Our use of ultrasonography and DXA allows for observations of the biceps brachii and examination of regional and total body composition changes in response to detraining and retraining. Ultrasound‐derived measurements of mCSA and DXA‐based assessments of lean mass have been compared against MRI and CT imaging, demonstrating good agreement and strong associations (Noorkoiv et al., 2010; Tavoian et al., 2019). However, the sensitivity of DXA in detecting longitudinal changes in lean mass appears questionable (Tavoian et al., 2019). We show that on average, mCSA of the biceps brachii for CON changed by −5% and +4.5% after Phase 1 and Phase 2, while the changes in TRAIN were ~+1.0% and ~+8%, respectively. The changes in cEI for CON following Phase 1 are similar to immobilization‐induced changes for the knee extensors (MacLennan et al., 2020). Similar patterns with smaller magnitudes are seen through the DXA data with regional changes of lean mass for CON at −2.8% and +2.7%, and +1.1 and +4.6% for TRAIN. Notably, total body composition changes differed between groups, with total body fat decreasing in TRAIN compared to CON after Phase 2. The directional changes and effect sizes support the effects seen by others with larger sample sizes (Andrushko et al., 2018). Our novel observations of body composition changes in response to arm slinging with or without concomitant unilateral resistance training are interesting to consider with regard to the changes that would occur with more profound or prolonged scenarios of muscle disuse and inactivity and offer further support for previous contentions that, at minimum, cross‐education protects against global deconditioning (Farthing & Zehr, 2014).

Our novel finding of enhanced task‐specific strength recovery in the immobilized arm as a result of cross‐education may be due to several factors. Most simply, the reduced strength loss after immobilization results in less weakness to overcome during retraining. As shown by Chen et al. (2023), eccentric‐emphasis training in the non‐immobilized arm reduces muscle damage in the immobilized arm after immobilization. These findings highlight the potential of eccentric‐emphasis cross‐education exercises to offer a protective effect during both immobilization and retraining. Additionally, the neural mechanisms underlying cross‐education must be considered. The bilateral‐access hypothesis and the cross‐activation hypotheses (Carroll et al., 2006; Ruddy & Carson, 2013) are the dominant, non‐mutually exclusive theories implicated. The details of these hypotheses have been discussed extensively elsewhere (Calvert & Carson, 2022). Regarding the present data, it is unclear how the attenuation of muscle atrophy may be explained solely through neural mechanisms, particularly given that mirror activity in the inactive homologous limb does not appear to be related to the cross‐education effect (Andrushko et al., 2017; Ruddy et al., 2016). Unfortunately, we did not measure EMG activity during training sessions to contribute to this debate. Cross‐education is known to be muscle group‐specific (Andrushko et al., 2017), which would seemingly rule out a systemic mechanism, as a systemic effect should result in non‐specific transfer. Therefore, it is plausible that cross‐education favorably preserves muscle protein synthesis pathways and/or decreases ubiquitination through a currently unknown mechanism (Hendy et al., 2012; Hendy & Lamon, 2017). Although this notion is challenged by other unilateral resistance training paradigms *preceding* lower limb disuse, showing the muscle atrophy sparing effects of resistance exercise are (Smeuninx et al., 2025) or are not (Jameson et al., 2021) confined to the exercised limb. Similarly, findings from animal models of disuse *concurrent* with unilateral mitigation strategies show ipsilateral (Lawrence et al., 2020) and contralateral (Miller et al., 2018) preservations of muscle size, with increases in muscle protein synthesis and turnover for the involved *and* uninvolved limbs. There is a clear need, therefore, to pursue the mechanisms underlying the attenuation of neuromuscular dysfunction with unilateral resistance training during disuse, as context‐ and sex‐specific responses may reveal novel therapeutic targets with broad implications.

## 5 LIMITATIONS

Several limitations must be considered when interpreting our data. Most notably, the small sample size of this pilot study and the lack of omnibus statistical approaches prevent definitive conclusions. Despite recruitment efforts over 2 years, we were unable to meet our enrollment goal due to the laboratory's relocation. Nevertheless, these preliminary findings, in females, provide valuable insights into the detraining–retraining timeline and can inform larger clinical trials and future meta‐analyses. This data has limitations in inferring neural mechanisms, as we collected surface EMG only from the biceps brachii. While this approach enables the assessment of global muscle excitability during elbow flexion, it does not provide information on specific motor unit behavior or upstream neural adaptations. Additionally, the absence of EMG monitoring for inactive homologous muscles during training, which could clarify the role of physiological mirror activity in the observed effects, is a notable limitation. The challenge of blinding in exercise interventions is well documented (Boutron et al., 2007). Although we blinded outcome assessors and attempted to mask the study hypotheses from participants, potential influences such as demand characteristics, expectancy effects, and placebo effects cannot be ruled out. Finally, while participants self‐reported their nutrition and physical activity habits and were instructed to maintain their usual routines, this information was collected only at testing time points and not objectively quantified. This may partly explain the observed body fat changes between groups, independent of the intervention.

## 6 CONCLUSIONS

The results of this pilot study demonstrate that unilateral resistance training of the uninvolved arm during 4 weeks of orthopedic immobilization preserves maximal muscle strength in the immobilized arm and likely facilitates task‐specific strength recovery during retraining. The absence of an effect on isometric elbow flexor and handgrip strength likely underscores the specificity of the cross‐education effect. These findings have implications for translational work aiming to identify mechanistic pathways and targets responsible for the preservation effects seen here and elsewhere for the immobilized arm as a result of unilateral resistance training.

## FUNDING INFORMATION

This study was supported by Texas Christian University's Research and Creative Activities Fund Award.

## CONFLICT OF INTEREST STATEMENT

The authors declare no conflicts of interest.

## ETHICS APPROVAL

This study received ethical approval from Texas Christian University's Institutional Review Board for Human Subjects Research (ID#2021–101) and all subjects signed an Informed Consent document.

## Supporting information


Appendix S1.

## Data Availability

Data are stored in a public repository with access through the corresponding author.
