# Supplementary material for: Cross‐education attenuates muscle weakness and facilitates strength recovery after orthopedic immobilization in females: A pilot study
Source: Physiol Rep. 2025 Apr 26;13(8):e70329. doi: 10.14814/phy2.70329 (PMC12032444; doi:10.14814/phy2.70329)
Supplement: Supplementary file 1 — Appendix S1. [file PHY2-13-e70329-s001.docx]

**Supplementary File 1.**

**Expanded Methodological Details**

**Orthopedic Immobilization**

Immobilization began immediately after baseline testing for each participant. Each participant was custom-fitted with a sling and swathe by the research team, ensuring the arm was comfortably restrained at 90 degrees of elbow flexion. Participants were then instructed on how to independently put on and remove the sling and swathe, practicing the technique that best suited their comfort and mobility. The research team provided strategies to help participants manage daily tasks with one hand or arm, including how to wear and adjust a backpack, groom, cut food with one hand, navigate doors, get up from the ground, and even how to safely fall. Participants were instructed to treat the immobilized arm as though it were genuinely injured, wearing the sling as often as possible for a minimum of 10 hours per day. To promote compliance and personalization, participants were permitted to decorate their sling and swathe with badges, emblems, or other decorations of their choosing.

To mitigate potential adverse effects, participants were instructed to perform approximately three minutes of daily shoulder mobility exercises. These exercises, demonstrated through online videos provided by the research team, focused on achieving a full range of motion in a supine position, including shoulder extension, flexion, and internal and external rotation, to prevent complications such as frozen shoulder. Each participant was assigned a safety officer who communicated with them via their preferred method of contact. The safety officer conducted regular check-ins to assess well-being, remind participants to complete their daily mobility exercises, and verify the number of hours the sling was worn each day. During the resistance training intervention, participants in the TRAIN group wore the sling during the Phase 1 training sessions.

At the conclusion of the study, each participant received a $400 USD Amazon gift card as compensation for their participation. Participants only received compensation if they completed the study in its entirety with no significant deviations from the protocol or intervention.

**Training Intervention**

*Familiarization*

All participants underwent a standardized familiarization and education process to ensure consistent coaching, movement cueing, and instruction. Participants were systematically introduced to the equipment, including dumbbells, clips, and weights, and instructed on proper grip, form, coordination, and safe weight management techniques. Familiarization involved practicing movements with a small, unweighted dumbbell (~2.2 lbs) until proper form was achieved. Subsequently, participants used an adjustable 10-lb dumbbell (Rogue DB25-10, 25mm diameter) for five repetitions as a joint-specific warm-up. This process was repeated with a weight they felt was approximately 50% of their maximal strength, allowing participants to gain confidence and competence with both the biceps curl and shoulder press exercises for both arms.

This study included two resistance training periods: Phase 1, exclusive to the TRAIN group, and Phase 2, where both TRAIN and CONTROL groups completed resistance training. In Phase 1, the TRAIN group performed eight unilateral resistance training sessions, while in Phase 2, both groups completed eight bilateral resistance training sessions. All sessions were supervised by a Certified Strength and Conditioning Specialist (CSCS), and each participant was matched with their safety officer who provided consistent guidance and continuity throughout the study. The training interventions included dumbbell biceps curls and shoulder presses, starting at 75% of each participant’s one-repetition maximum (1RM). Each session comprised three to five sets of five repetitions, with progressive overload implemented by increasing the number of sets to five during the first three sessions of each phase (Table below). Intensity was adjusted as tolerated, and volume was reduced to three sets for the final two sessions of each phase to minimize muscle soreness and edema. Training occurred twice weekly, with at least 48 hours between sessions, and was scheduled at approximately the same time of day (± 1 hour) for each participant. Familiarization and education procedures, along with detailed training parameters for both phases, are described below.

| **Table 1. The number of sets, repetitions, and intensity progressions for each respective training visit and exercise during Phase 1 as well as Phase 2.** | | | | | | | | |
| --- | --- | --- | --- | --- | --- | --- | --- | --- |
| **Training day No.** | 1 | 2 | 3 | 4 | 5 | 6 | 7 | 8 |
| **Set No.** | 3 | 4 | 5 | 5 | 5 | 5 | 3 | 3 |
| **Repetitions per set** | 5 | 5 | 5 | 5 | 5 | 5 | 5 | 5 |
| **Intensity progression if capable and tolerated (+)** |  |  | + | + | + | + | + | + |

*Phase 1*

Phase 1 training was exclusive to the TRAIN group and focused on unilateral training of the right, non-immobilized arm. Before each session, participants performed a five-minute brisk walk on a self-propelled treadmill as a general warm-up. Training sessions took place individually in a dedicated laboratory training space equipped with emergency response resources, including an AED. Each session was supervised by at least two research team members, one of whom held a CSCS or Personal Training certification, to assist with data recording and loading of the dumbbells.

Participants had autonomy over music selection and volume during sessions to enhance motivation. Following the warm-up, participants performed a joint-specific warm-up comprising two sets at light loads (e.g., unweighted dumbbell and 50% of 1RM). Participants were also permitted to perform additional stretching or mobility exercises as needed. Lifting chalk and hair ties, including ponytail holders, were available during training to enhance participant comfort during training.

*Training Protocol*

The biceps curl was performed in a standing position, with weights loaded by a researcher and placed on a commercially available weight bench for participant retrieval. The shoulder press was conducted on an adjustable bench inclined to approximately 85 degrees. Weights were handed to the participant at shoulder level to initiate the exercise and retrieved similarly upon completion.

Training intensity began at 75% of 1RM, starting with three sets of five repetitions on the first session. Subsequent sessions increased to four and then five sets. Intensity progression was based on participants’ ability to complete prescribed volumes, movement speed, perceived exertion (measured via the OMNI-RPE scale), and overall comfort. Microplates (increments of 0.25 lbs) facilitated precise and consistent load adjustments. Participants were instructed to stop sets before they reached momentary failure, allowing for short rest intervals (30 seconds to 1 minute) if needed to complete prescribed repetitions. Recovery intervals between sets and exercises were standardized at two minutes and monitored using a timer. Participants were instructed to perform the exercises without relying on body momentum, maintaining control of the weight at their preferred tempo.

To prevent lingering fatigue or soreness that might affect post-testing measures, volume was reduced to three sets of five repetitions during the final two sessions. Each session was comfortably completed in approximately 30 minutes.

*Adverse Events and Adherence*

No adverse events occurred during training. All planned sessions were completed as outlined, except for one participant who withdrew due to an unrelated respiratory illness after two sessions. The intervention demonstrated high adherence (100%) among the remaining participants and was delivered as intended, with no deviations from the protocol.

**Phase 2**

The training intervention during Phase 2 adhered to the same format, procedures, and outline as Phase 1, with the inclusion of the CONTROL group and training of the left, previously immobilized arm. Training commenced 2-3 days after the Phase 1 post-testing visit. The measured unilateral 1RM for each arm obtained during Phase 1 post-testing guided the starting intensity for Phase 2. Training began at approximately 75% of the measured 1RM for each arm, resulting in participants handling slightly different weights for their arms initially. While this asymmetry was briefly challenging, participants quickly adapted with practice, aided by researchers who managed weight placement and retrieval during biceps curl and shoulder press exercises, as described for Phase 1.

Just as during Phase 1, participants began with three sets of five repetitions, progressing to five sets of five repetitions with incremental increases in intensity as tolerated (See Table 1). All other procedures, including motivation strategies, programming, and warm-up protocols, mirrored those from Phase 1 to maintain consistency and standardization.

Upon completing each training session, participants consumed a protein dose of ~0.2 g per pound of body weight using lactose-free cow’s milk (Fairlife Whole Ultra-Filtered Milk, Fairlife, Inc., Chicago, IL). The amount of milk ranged from 7 to 12 ounces, depending on individual body weight. Milk was stored in a laboratory refrigerator, measured by a researcher, and dispensed in single-use disposable cups. Participants consumed the entire dose under researcher supervision before leaving the laboratory. This recovery protocol was exclusive to Phase 2 to optimize muscle recovery following immobilization.

*Adverse Events and Adherence*
No adverse events occurred during Phase 2. All participants completed the prescribed eight sessions (100%). Minor deviations included scheduling adjustments when participants needed to reschedule due to unforeseen conflicts, such as impaired sleep or weather-related issues. These adjustments ensured all training sessions were completed with sufficient recovery time. Other minor deviations involved participant attire; some participants attended in professional clothing due to interviews but remained fully engaged in the training sessions.

**Exploratory Analyses**

Exploratory regression analyses were conducted to examine potential associations between strength gains and morphological adaptations in the previously immobilized arm during the retraining period for all participants. Specifically, we analyzed the relationships between relative changes in biceps curl one-repetition maximum (1RM) and biceps brachii muscle cross-sectional area (mCSA), biceps curl 1RM and DXA-derived regional lean mass, and shoulder press 1RM and DXA-derived regional lean mass (n = 10). These analyses aimed to identify the morphological factors potentially contributing to strength improvements. The absence of significant associations between these variables suggested that the observed strength gains were predominantly attributable to neural adaptations rather than structural changes in the muscle tissue. The scatterplots are shown in Supplementary Figure 1.

***
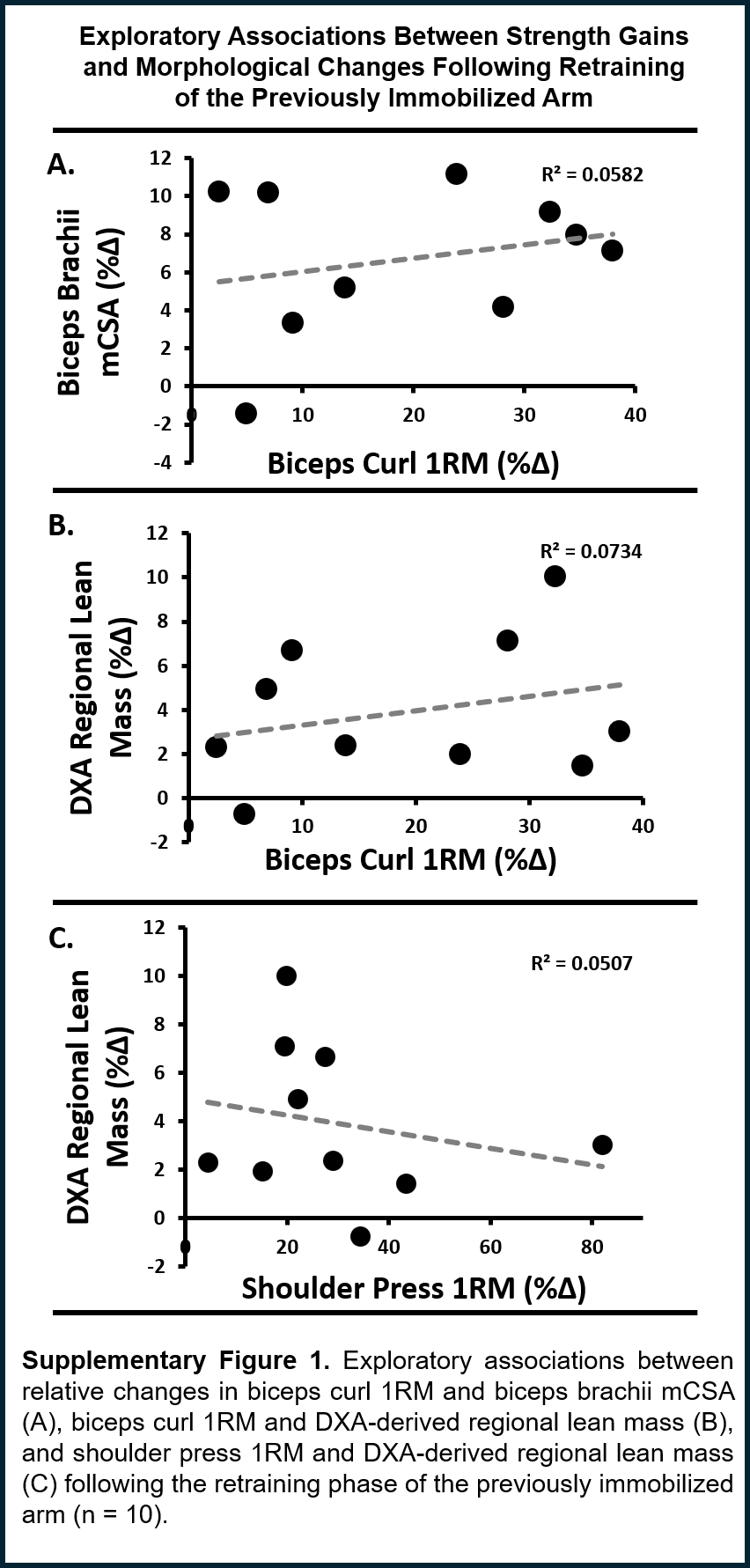
***
